# The multidimensional relationship between alpha oscillations and cognition

**DOI:** 10.1162/IMAG.a.96

**Published:** 2025-08-04

**Authors:** Agatha Lenartowicz, Sebastian C. Coleman, Nicolas Zink, Karen J. Mullinger

**Affiliations:** Department of Psychiatry & Biobehavioral Sciences, OneMind Staglin Center for Cognitive Neuroimaging, Semel Institute for Neuroscience and Human Behavior, University of California, Los Angeles, CA, United States; Sir Peter Mansfield Imaging Center, School of Physics and Astronomy, University of Nottingham, Nottingham, United Kingdom; Neurosciences & Mental Health, SickKids Research Institute, Toronto, ON, Canada; Center for Human Brain Health, School of Psychology, University of Birmingham, Birmingham, United Kingdom

**Keywords:** alpha, oscillations, attention, excitability, synchronization

## Abstract

Alpha oscillations are a robust neurophysiological phenomenon associated with cortical suppression and synaptic input gating, functionally interpreted as a mechanism of selective attention. Here, we highlight known dissociations between alpha oscillations and selective attention that question the specificity of this interpretation. We postulate that the inconsistencies are accounted for when we consider alpha oscillations as a neurophysiological mechanism that tracks cortical excitability, but one that can be modulated by a multitude of factors that include but are not limited to selective attention and include bottom-up and top-down interactions, internal processes, and regulatory system influences on cortical excitability. Thus, reverse inference regarding the cognitive role of alpha modulations may depend on experimental context. Importantly, this perspective reiterates that there exists a significant need for research that disentangles the mechanistic bases of alpha oscillations across different cognitive phenomena.

## 1 Alpha Oscillations as a Substrate of Selective Attention

The alpha rhythm is an 8–12 Hz neural oscillation (Berger, 1929), ubiquitous to brain function (documented across cat, dog, primate, rat, guinea pig, and human) (Steriade et al., 1990). The first comprehensive functional framework of alpha oscillations invoked an “idling” cortical system (Pfurtscheller et al., 1996a) (Table 1), *neither receiving nor processing sensory information*, based on greater alpha power in disengaged states, attenuated with cortical engagement. The turn of the 21^st^ century saw a theoretical shift in alpha research, dominated by study of alpha power modulations during transient cognitive events (e.g., stimulus encoding) relative to a non-rest baseline period, that recast the function of alpha as a gating mechanism in the service of selective attention and memory (Foxe & Snyder, 2011; Jensen & Mazaheri, 2010; Klimesch, 2012). Supporting this idea, alpha power increases were reported to coincide with weaker processing of sensory inputs (e.g., Iemi et al., 2022), while decreases with stronger cortical excitability (e.g., Lange et al., 2013). Alpha oscillations were proposed to inhibit cortical processing (Mathewson et al., 2011; Mazaheri & Jensen, 2010), with phase supporting information sampling (also Fiebelkorn & Kastner, 2018). Additionally, laminar (e.g., Bollimunta et al., 2011), functional connectivity (e.g., Lobier et al., 2018), and causal neurofeedback (e.g., Bagherzadeh et al., 2020) or neuromodulation (e.g., Capotosto et al., 2009) studies further identified a role for slow-frequency oscillations in inter-areal synchronization and, by extension, a role for alpha oscillations as a carrier of modulatory signals that gate information flow in sensory cortex (Bonnefond et al., 2017; Fiebelkorn & Kastner, 2018; Palva & Palva, 2011). This body of research helped to cement the implied role of alpha in implementation of selective attention, the impact of which is evident in real-world alpha-based monitoring, from vigilance and drowsiness (Wang et al., 2014), to stress (Ahn et al., 2019), to neurofeedback in ADHD (Mishra et al., 2021). Yet, the last decade of research has also revealed inconsistencies in empirical effects in alpha versus selective attention that question the specificity of its role. Here, we recapitulate these inconsistencies and, considering these data, postulate that while alpha oscillations track dynamic changes in cortical excitability, these subsume selective attention as one of *multiple contributing factors*. This subtle shift from a functional to neurophysiological perspective on alpha oscillations offers one account for the empirical inconsistencies and highlights the need to mechanistically define the many instantiations of alpha.

**Table 1.** Neurocognitive frameworks of alpha oscillations.

		Explanatory Focus			
Model	Key Tenets	α *↑*	α *↓*	ɸ	FC
**Intrinsic oscillations**(Lopes da Silva, 1991; Lopes da Silva et al., 1976)	Intrinsic oscillations with generators in cortical layer 5 and thalamus, but functionally distinct from thalamic sleep spindles. Putative functions include gating of information flow and/or mediation of neuronal assemblies.	x			
**Idling rhythm**(Pfurtscheller & Lopes da Silva, 1999; Pfurtscheller et al., 1996a)	Alpha rhythm represents cortex in idling or “nil-working” state, possibly resulting in interruption of thalamo-cortical information transfer.	x			
**Inhibition-timing hypothesis**(Klimesch, 2012; Klimesch et al., 2007)	Alpha increases associated with cortical suppression; decreases accompany information storage and retrieval; phase is a mechanism for information selection.	x	x	x	
**Attentional gating by inhibition**(Jensen, 2023; Jensen & Mazaheri, 2010; Mathewson et al., 2011)	Alpha increases reflect cortical inhibition, a mechanism for information routing, with inhibition occurring at alpha peaks, producing pulsed inhibition dynamics. Alpha may be triggered by competitive dynamics due to target processing	x		x	
**Suppression mechanism**(Foxe & Snyder, 2011)	Alpha increases play a role in distractor inhibition in selective attention.	x		x	
**Information by desynchronization**(Hanslmayr et al., 2012)	Alpha decreases are proportional to amount of information in input signal.		x		
**Inter-regional & network coordination**(Bonnefond et al., 2017; Palva & Palva, 2007, 2011)	Alpha band inter-areal synchrony supports active processing in long-range networks (e.g., fronto-parietal network).	x		x	x
**Sustained attention hypothesis**(Clayton et al., 2015)	Alpha increases serve in cortical inhibition. Low-frequency (theta and alpha) phase synchronization supports long-range transmission of information.	x		x	x
**Substrate of cognitive control**(Sadaghiani & Kleinschmidt, 2016)	Alpha increases recruit cingulo-opercular network and clearing of information; decreases recruit dorsal attention network and selective attention; phase inter-areal synchronization associated with fronto-parietal network and adaptive control.	x	x	x	x
**Rhythmic theory of attention**(Fiebelkorn & Kastner, 2018)	Alpha phase supports inter-areal communication for the sampling of information; power decreases facilitate high-frequency firing in selection of information.			x	x

Note. α ↑ = alpha power increase, α ↓ = alpha power decrease, ɸ = alpha phase, FC = functional connectivity. Rows organized by explanatory focus: alpha power increases and generation (top), bidirectional alpha power modulations and phase effects (middle), inter-regional communication (bottom).

## 2 Empirical Inconsistencies

**Fig. 1.**
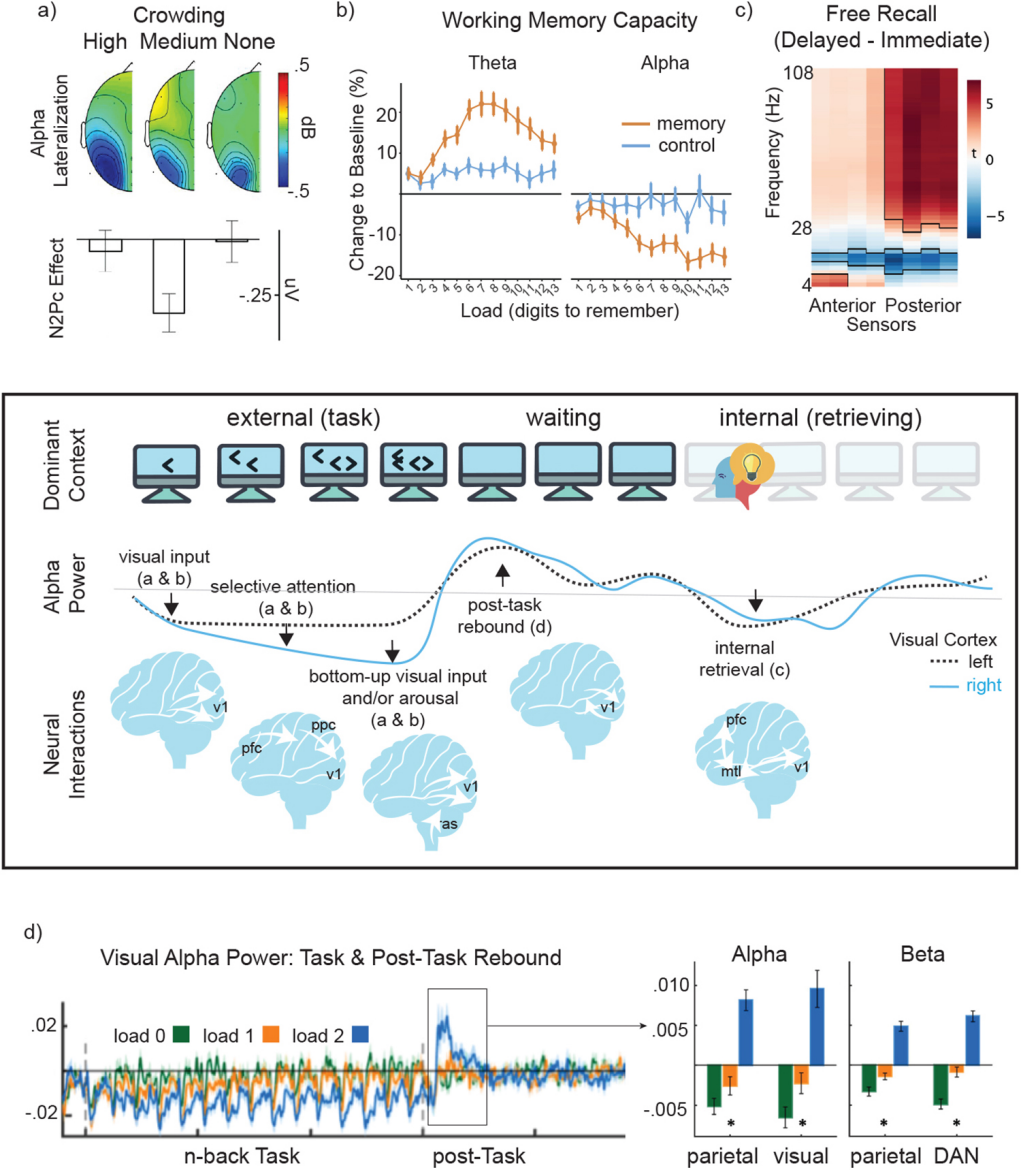
Alpha inversely tracks cortical excitability across multiple sources. Examples of alpha power dissociates from selective attention: (a) lateralized decreases of alpha power with spatial attention (top, blue) that increase with visual crowding, unlike the N2Pc (bottom), a feature of selective attention maximal at medium crowding (Bacigalupo & Luck, 2019); (b) alpha power decreases as memory load increases, unlike theta (4–8 Hz), which plateaus at memory capacity (Kosachenko et al., 2023); (c) alpha (and beta) power (blue) decreases more after a delay than during immediate free recall of episodic items (Katerman et al., 2022); and (d) alpha power decreases *during* task are followed by involuntary increases *after* the task (Coleman et al., 2023) (i.e., post-task rebound), consistent with post-task self-organization. Middle panel shows hypothetical dynamics of how such sources of alpha modulation could arise during spatial attention, along with putative neural interactions (Desimone & Duncan, 1995). Namely, alpha power decreases across visual cortices (v1) with stimulus onset (thalamic inputs), more strongly in right hemisphere following a selective attention instruction and fronto-parietal (pfc-ppc) bias (producing a lateralization effect like in (a) & (b)), then continues to decrease in target-relevant cortex with stimulus complexity, reflecting effects of bottom-up visual inputs or arousal (ras = reticular activating system) (like in (a) & (b)). Following task, alpha power rebounds via local self-regulatory interactions (like in (d)). Subsequently, an internally generated memory (e.g., retrieved via medial temporal lobe interactions, mtl) triggers another alpha power decrease (like in (c)), further highlighting how reference choice may affect interpretation of alpha power modulations. Figure is modified and reproduced as per Create Commons License.

While alpha modulations often correlate with effects of selective attention, a growing body of data suggests that event-related modulations of (primarily occipital) alpha power does not always track selective attention effects—namely the enhancement of neural responses during attended stimuli versus ignored stimuli. For example, in an auditory selective attention task (Bonacci et al., 2020), experimenters manipulated the availability of spatial versus pitch information when responding to an attended auditory stimulus. A selective attention effect (attended > ignored) was present regardless of such information in an early event-related potential (ERP) known as N1. The same effect in event-related decreases (ERDs) of alpha power relative to pre-stimulus baseline was absent when non-spatial pitch information was available for selective attention, leading the authors to suggest that alpha power tracks *spatial* orienting in selective attention, when present. In another study (Bacigalupo & Luck, 2019), alpha ERDs were compared with that of another ERP, N2Pc, an indicator of target processing. The N2Pc, but not alpha ERD, was sensitive to distractor-generated competition in the visual field, whereas alpha ERD monotonically decreased as task difficulty increased, which lead the authors to suggest that alpha tracked effort not attentional selection (Fig. 1a). Several studies have also reported dissociations between alpha ERDs and attention effects in steady state visual evoked potentials (SSVEPs) that track sensory cortical responses (Antonov et al., 2020; Gundlach et al., 2020; Zhigalov & Jensen, 2020). Adding to these dissociations, in studies that independently manipulated target versus distractor stimuli, event-related alpha-power increases (ERIs) during distractor processing were reported as inconsistent (Noonan et al., 2016; Wostmann et al., 2019), or a side-effect of target processing (Gutteling et al., 2022). In complement, in a review of 100 working memory studies (Pavlov & Kotchoubey, 2020), only 60–80% showed expected ERIs in alpha power during maintenance, when distractor-related control over cortex would be predicted (also see Lenartowicz et al., 2021).

In addition, several studies report a mismatch in the temporal dynamics of alpha and selective attention processes. In the above-noted study of alpha and N2Pc (Bacigalupo & Luck, 2019), attention effects on alpha power ERDs lagged those in the target-sensitive N2Pc for two of three conditions tested. In one study of SSVEPs, the onset of attention effects in alpha power had a 130–180 ms lag relative to those in behavior and SSVEPs (Antonov et al., 2020), whereas in another a mean lag of 176 ms was reported (Gundlach et al., 2020). Differences also exist on longer time scales, in time-on-task effects. Ferrante et al. (2023) varied distractor probability, inducing learning effects and corresponding downregulation of high-frequency SSVEP response for high-probability distractors, but this effect was not present in concurrently measured alpha ERDs. Similarly, Kosachenko et al. (2023) tracked time-on-task effects as 2 to 15 stimuli were sequentially presented to participants for subsequent recall. Pupil size and oscillatory power in the theta band (4–8 Hz) increased with increasing load, reached a plateau, and proceeded to decline, consistent with attention disengagement due to memory capacity overload. However, this pattern was not tracked by alpha power ERDs, which instead decreased monotonically with increasing number of stimuli in sequence (Fig. 1b).

Finally, several studies have reported alpha power modulations in the absence of attention modulations, not considered in frameworks of selective attention. In a 2008 study, participants viewed initially distorted images of objects that gradually increased in clarity (Freunberger et al., 2008). The onset of alpha power ERD coincided with the moment of object recognition, with no notable change in attention. More recently, alpha dynamics were studied during spontaneous free-recall of previously learned words after either a few seconds or several days (Fig. 1c) (Katerman et al., 2022). Alpha and beta decreased preceding spontaneous verbal responses of remembered words, again with no apparent manipulation of attention. The effect is not specific to memory. Alpha decreases arise during math problem solving (Brunner et al., 2021), and dynamic internally-generated shifts of attention (i.e., free of stimulus-driven or motor effects) (Peylo et al., 2024), again in the absence of attention manipulation. In sum, current data suggest that alpha modulations can be absent where attention varies, temporally lags, or deviates from attention effects, and can vary when attention does not.

## 3 Alpha Oscillations Track Multiple Influences on Cortical Excitability

The data suggest that alpha power modulations cannot be ubiquitously interpreted as modulations of selective attention. Similar concerns have been raised by others considering specifically the inconsistencies in evidence for alpha’s relationship to suppression (Foster & Awh, 2019), as well as degree and causality of relationship to evoked-responses (Morrow et al., 2023; Peylo et al., 2021). Here, we consider the problem from another perspective, asking why alpha power sometimes tracks attention effects and sometimes it does not, yet reliably couples to cortical excitability (Lange et al., 2013; Sauseng et al., 2009). In particular, the synchronous rhythmic neural spiking that defines the presence of alpha range oscillations is postulated to affect the probability of that cortex to generate action potential response given an input signal, thus its “cortical excitability” (Fiebelkorn & Kastner, 2018; Klimesch, 2012; Miller et al., 2024; Yuasa et al., 2025). While this definition might at first glance suggest that changes in cortical excitability (and alpha power) should reliably accompany the onset of selective attention effects (when cortex is more or less active), dissociations become feasible when we consider that alpha power recorded by an electrode at the scalp or cortical surface is sensitive to several different factors that influence cortical excitability, not just those that contribute to selective attention effects.

For instance, alpha power varies as a function of physiological arousal (e.g., as induced by caffeine) (Barry et al., 2005) and fluctuates spontaneously in recorded signals (Sadaghiani & Kleinschmidt, 2016), an effect proposed to correspond to changes in overall responsivity (not discriminability) (Samaha et al., 2020), akin to shifts in baseline cortical excitability. Similarly, alpha power can decrease with increasingly complex sensory input as a broader area of cortex is engaged in processing, or due to interactions between a neighboring region of cortex and some other part of the brain as observed during semantic or memory retrieval (Klimesch, 2012). In principle, careful experimental design would control for such a multitude of factors in selective attention paradigms. Namely, comparing a stimulus response relative to a baseline as is done in event-related effects (ERDs or ERIs) or to another condition (e.g., attend minus ignore) should isolate only the effect of interest. However, this is not necessarily the case if such alternate factors affect one condition differentially than the other. As shown in Figure 1 (middle panel), increasing visual sensory input may affect cortical excitability in target visual cortex (i.e., “attend”) differentially than in non-target visual cortex (i.e., “ignore”) contributing to the appearance of a selective attention effect. Moreover, selective attention indices (e.g., event-related measures for attended versus ignored stimuli) need not reflect activity in the exact same neural population as those captured by alpha power, both are distant from the source and reflect mixed signals from the perspective of an electrode, and thus may be subject to different sensory or cortico-cortical interactions affecting signal variance (Klimesch, 2012).

As such, alpha power may be sensitive to different factors in different task contexts (Fig. 1), contributing to the reviewed empirical inconsistencies. In particular, the dissociations between validated metrics of selective attention, such as the N2Pc or enhanced sensory signals for attended versus ignored stimuli (e.g., in SSVEPs or the N1), suggest that whereas the former metrics faithfully track selective attention effects during sensory processing, alpha power may also index visual cortex activation due to, for instance, spatial orienting, such as during pitch-based selective attention as discussed by Bonacci et al. (2020) (also see Wildegger et al., 2017). Another factor, considering its monotonic responses to greater stimulus crowding (Bacigalupo & Luck, 2019) (Fig. 1a) or memory load (Kosachenko et al., 2023) (Fig. 1b), may be increased excitability across visual cortex due to spatially complex or temporally sustained visual input. The numerous dissociations between alpha power and SSVEP effects on early sensory processes, and lagging effects of selective attention on event-related alpha power, have also been argued to reflect alpha being less sensitive or noisier as a metric (Morrow et al., 2023; Peylo et al., 2021), but could, in complement, be dominated by post-sensory processes which would be likely to engage broader cortex than early sensory modulation, or different sources altogether, such as post-sensory cortex (e.g., occipito-parietal or parietal) as proposed by some (Gundlach et al., 2020; Sokoliuk et al., 2019; Zhigalov & Jensen, 2020). A further possibility is that sensory selective attention effects in alpha power in these studies are absent because stimuli produce both increases in broadband power and also a decrease in alpha power, leading to cancelation effects when not modeled separately (Yuasa et al., 2025).

Finally, the known relationship between alpha power and cortical excitability may also help explain the variability in alpha power during working memory maintenance across paradigms (Lenartowicz et al., 2021; Pavlov & Kotchoubey, 2020), because such paradigms vary in stimulus content, which may determine whether visual cortex—a prime contributor to occipital alpha signals—is engaged or disengaged during maintenance. Consistent with this prediction, Pavlov and Kotchoubey (2020) report that alpha power increases are more reliable in paradigms where content is verbal (78% show alpha increase) versus visual (57% show alpha increase), which is consistent with visual cortex being less likely to be involved in the maintenance of verbal than visual stimuli. In complement, repeated 10-Hz transcranial magnetic stimulation to superior parietal lobe during visual working memory maintenance produces a negative correlation between alpha power change and accuracy change during encoding of spatial (location) but not object content, with the former but not latter thought to recruit the stimulated cortex for storage processes (Hamidi et al., 2009). This is consistent with reported specificity of alpha-range stimulation in parietal cortex to endogenous visuospatial attention (Kemmerer et al., 2022).

A notable benefit of considering alpha as a readout of cortical excitability is that it helps to bridge effects in selective attention with modulation in alpha power outside of selective attention paradigms, such as reports of alpha power decreases during free-recall (Katerman et al., 2022) (Fig. 1c) and mathematical problem solving (Brunner et al., 2021), among other examples of *internally willed attention* (Lenartowicz et al., 2013; Nadra et al., 2023). Such modulations are consistent with cortical excitability in visuo-parietal networks triggered by cortico-cortical interactions, echoing the proposed role of alpha power in information retrieval from the brain’s storage systems (Klimesch, 2012) (Table 1). Of note are eyes-closed paradigms, such as in *interoceptive attention* (e.g., breath-counting), in which alpha power decreases have been reported during episodes of mind-wandering (Braboszcz & Delorme, 2011) and increases during low-consistency performance (Ramanathan et al., 2024), which highlights that internally focused processes, and thus cortical excitability in visual cortex, can fluctuate due to internally generated cortical interactions, rather than external sensory input (Ramanathan et al., 2024; Sadaghiani & Kleinschmidt, 2016). Finally, this perspective accommodates observations of alpha power effects in *internally generated but task-unrelated* neural processing, such as time-on-task effects (Kopčanová et al., 2025) or low-frequency (0.01–0.1 Hz) relationships to global signal changes in fMRI studies (Bolt et al., 2024), demonstrated to covary with changes in autonomic system dynamics (Bolt et al., 2024; Xu et al., 2025). An interesting phenomenon in this category is the oscillatory rebound, defined as a power increase (relative to rest) following cessation of task activity (Pfurtscheller et al., 1996b) (e.g., Fig. 1d). Though primarily been studied in the beta band (13–30 Hz) in sensorimotor areas (Jurkiewicz et al., 2006), both alpha and visual beta rebounds have been reported (Coleman et al., 2023; Mullinger et al., 2013). The rebound appears to reflect a self-regulatory processes in networks undergoing an abrupt reduction in cognitive load (Chen et al., 1998), reminiscent of the idling hypothesis of alpha oscillations proposed in the 1990s (Pfurtscheller et al., 1996a) (c.f., Table 1), or, of “self-organization” as may be needed for rebalancing of excitatory and inhibitory interactions (Buzsaki & Draguhn, 2004). Thus, alpha power tracks changes in cortical excitability both during externally driven selective attention tasks as well as in internally generated phenomena.

Notably, while we postulate that alpha power may be better conceptualized as a read-out signal on cortical excitability than a biomarker of selective attention, this does not invalidate the presence of selective attention effects in alpha power, its role in facilitating neural responses during attending (Mishra et al., 2012), or the value of existing models in explaining alpha’s role (be it power or phase) in selective attention phenomena (Table 1). Rather it suggests that the explanatory strength of such models may not encapsulate the breadth of the phenomena that can contribute to the recorded changes in alpha power at an electrode. Systematic assessment of how such multiple contributing factors interact and how they can be dissociated is an important research goal (also see Morrow et al., 2023; Peylo et al., 2021; Samaha et al., 2020). The presence of multiple effects in the reviewed studies suggests that quantifying alpha across multiple time windows along with other indicators of selective attention (e.g., Bacigalupo & Luck, 2019), and in reference to baselines other than the typical pre-stimulus interval with adoption of continuous paradigms (e.g., Peylo et al., 2024), may be particularly valuable in helping to dissociate contributing factors. Indeed, some of the reviewed studies on dissociations between alpha power and selective attention report not a complete absence of selective attention effects in alpha but, rather, the presence of *both* a selective attention effect (e.g., lateralization of alpha with spatial attention) and, also, a “non-attentional” effect (e.g., increased alpha power effect with number of stimuli). This represents an opportunity (and a paradigm) to understand these contributions.

## 4 Alpha Oscillations Gate Cortical Activity across Cognitive Phenomena

It is perhaps of note that the present analysis implies a change of perspective on the relationship between alpha and cognition more broadly, with emphasis on alpha serving a broad neurophysiological gating function across cortex and therefore supporting many cognitive constructs of which selective attention is only one. That is, as reviewed above, alpha oscillations are sensitive to effects of internally orienting sources (e.g., retrieval of memory or willed attention), bottom-up inputs (e.g., number of stimuli on screen), top-down effects (e.g., spatial attention effects on lateralization of alpha power), and cortical self-organization (e.g., rebounds).

The idea that alpha plays a gating function broader than for selective attention derives naturally from current oscillatory theory. Generators of alpha oscillations have been observed across the brain, consistent with multiple sources (also discussed in Peylo et al., 2021). Sources exist across visual, auditory, and somatosensory cortices (Haegens et al., 2015) as well as in associative cortex (e.g., parietal regions) (Sokoliuk et al., 2019), and subcortically within thalamus (Lopes da Silva, 1991). The presence of alpha oscillation across these structures is, thus, consistent with a gating role that is fast and dynamic. Indeed, within the laminar column, alpha generators have been documented across multiple layers (Bollimunta et al., 2011; Haegens et al., 2015), refuting earlier views that alpha generators are primarily in deep layers and carriers of so-called feedback signals (in contrast to faster oscillations, as carriers of feed-forward signals) (Buffalo, et al., 2011). Similarly, the propagation of alpha oscillations as traveling waves, postulated to arise through supra-granular, sparse-network connections (Davis et al., 2021; Muller et al., 2018), can change direction depending on behavioral state (Alamia et al., 2023; Pang 庞兆阳 et al., 2020). The direction can switch from backward to forward (posterior to anterior) when the behavioral state changes from quiet wakefulness to visual stimulation (Pang 庞兆阳 et al., 2020) or with stimulus relevance to behavioral goal (Zeng et al., 2024). Thus, alpha oscillations play a role in the gating and propagation of inputs across cortex, but do not necessarily have a dedicated functional role limited by direction of travel or a particular cognitive hierarchy.

Thus, both empirical cognitive effects and neurophysiological features of alpha oscillations would suggest that alpha power changes cannot be pinned to any single cognitive construct. It may be, however, that considering alpha as a distributed gating signal can inform cognitive theory. Given that dynamics of alpha oscillations are observed broadly across cortex and laminar interactions, perhaps attention may be better thought of as an emergent function (Perone et al., 2021; Postle, 2006; Zink et al., 2021), a product of distributed computations in the brain rather than a given network. Similar arguments have been made for the emergence of executive functions (Zink et al., 2021), working memory (Postle, 2006), and other complex cognitive functions (Miller et al., 2024; Pessoa et al., 2022) that have not been successfully localized to a unitary network. Some have suggested that it may perhaps be best to drop the term “attention” as a label for a specific functional system and instead focus on the many systems that implement defined sub-processes (Hommel et al., 2019). Regardless, the neurophysiological properties of alpha and its multidimensional relationship to cognition remind us that dynamics observed in selective attention paradigms reflect one constrained niche of computations within a dynamic, distributed system.

## 5 Conclusion

In sum, though modulation of alpha oscillation can be indicative of selective attention processes, such reverse inference may depend on experimental context. A more robust interpretation may lie in its neurophysiological role modulating cortical excitability across cortex, regardless of driving factors—that include bottom-up and top-down interactions, internal processes, and regulatory system influences on cortical excitability. There exists thus a pressing need to understand these relative contributions, their sources, and neurophysiological mechanisms, to fully capture the role of alpha oscillations in cognition.

## Data Availability

No code or data are associated with this manuscript.
